# The effects of experimentally induced hyperthyroidism on the diving physiology of harbor seals (*Phoca vitulina*)

**DOI:** 10.3389/fphys.2012.00380

**Published:** 2012-09-28

**Authors:** Gundula M. Weingartner, Sheila J. Thornton, Russel D. Andrews, Manfred R. Enstipp, Agnieszka D. Barts, Peter W. Hochachka

**Affiliations:** Department of Zoology, University of British ColumbiaVancouver, BC, Canada

**Keywords:** thyroid hormones, metabolism, hypometabolism, diving, hyperthyroidism, harbor seal, heart rate, lactate

## Abstract

Many phocid seals are expert divers that remain submerged longer than expected based on estimates of oxygen storage and utilization. This discrepancy is most likely due to an overestimation of diving metabolic rate. During diving, a selective redistribution of blood flow occurs, which may result in reduced metabolism in the hypoperfused tissues and a possible decline in whole-body metabolism to below the resting level (hypometabolism). Thyroid hormones are crucial in regulation of energy metabolism in vertebrates and therefore their control might be an important part of achieving a hypometabolic state during diving. To investigate the effect of thyroid hormones on diving physiology of phocid seals, we measured oxygen consumption, heart rate, and post-dive lactate concentrations in five harbor seals (*Phoca vitulina*) conducting 5 min dives on command, in both euthyroid and experimentally induced hyperthyroid states. Oxygen consumption during diving was significantly reduced (by 25%) in both euthyroid and hyperthyroid states, confirming that metabolic rate during diving falls below resting levels. Hyperthyroidism increased oxygen consumption (by 7–8%) when resting in water and during diving, compared with the euthyroid state, illustrating the marked effect of thyroid hormones on metabolic rate. Consequently, post-dive lactate concentrations were significantly increased in the hyperthyroid state, suggesting that the greater oxygen consumption rates forced seals to make increased use of anaerobic metabolic pathways. During diving, hyperthyroid seals also exhibited a more profound decline in heart rate than seals in the euthyroid state, indicating that these seals were pushed toward their aerobic limit and required a more pronounced cardiovascular response. Our results demonstrate the powerful role of thyroid hormones in metabolic regulation and support the hypothesis that thyroid hormones play a role in modulating the at-sea metabolism of phocid seals.

## Introduction

Diving mammals spend extended periods underwater, periodically returning to the surface to breathe. Under water, adjustments to their metabolic rate must occur in order to manage a limited supply of oxygen, while the accumulation of carbon dioxide and possibly lactate has to be tolerated. In the 1930's and 1940's Scholander and Irving described the most striking physiological alterations in avian and mammalian divers during forced submergence experiments: apnea, a profound slowing of heart rate (bradycardia), peripheral vasoconstriction, and accumulation of lactic acid (Irving, 1939; Scholander, 1940). Scholander referred to these responses as the “master switch of life,” because they were found not only in divers, but also in most vertebrates faced with asphyxia. These metabolic adjustments allowed for the reduction in whole-body aerobic metabolism and served as a mechanism to conserve the limited oxygen for hypoxia-sensitive tissues, such as the heart and brain (Scholander, 1963). To this day, these observations are accepted as the main components of the so-called diving response.

In the last few decades, however, technological development has enabled us to record behavioral and physiological variables in many species when voluntarily diving in their natural environment. From these studies, it has become apparent that the physiological responses during natural diving are much less pronounced than during forced submergence (for review see: Kooyman, 1985; Butler and Jones, 1997). In fact, most observations of freely diving animals indicate that the majority of dives are well within their aerobic dive limit (ADL), defined as the longest possible dive duration without an increase in blood lactate concentration (Kooyman, 1985). Hence, during routine diving, most divers might not require the extreme cardiovascular responses observed in forced submergence experiments. Some species, however (e.g., northern and southern elephant seals, *Mirounga angustirostris* and *Mirounga leonina*) remain submerged for periods longer than would be expected based on the estimates of available oxygen stores and metabolic rate (Le Boeuf et al., 1988; Hindell et al., 1992). A reduction in metabolism during diving to a level below resting (“hypometabolism”), or the partial reliance on anaerobic metabolism, or a combination of both have been proposed as physiological mechanisms to extend voluntary dive duration (Castellini, 1985; Fedak and Thompson, 1993). The latter authors investigated the behavioral and physiological consequences for a seal when using anaerobic metabolism to extend dive duration. The poor energy yield associated with anaerobic metabolism, combined with the accumulation of lactate make this metabolic process a costly endeavor. Consequently a seal would have to drastically increase the time spent at the surface following an anaerobic dive and/or be forced to conduct a series of short dives, well within its calculated ADL (Fedak and Thompson, 1993). While this might occur in some divers on rare occasions, anaerobic metabolism seems to contribute little energy during routine diving in endotherms (Butler, 2004). An alternative concept that could explain dive durations beyond the calculated ADL is hypometabolism. One central aspect of the diving response is the selective redistribution of blood flow during diving, possibly leading to hypometabolism in under-perfused tissues and a decline in whole-body metabolism. Although the mechanisms are still being investigated, regional hypothermia could be one potential way of achieving a reduced metabolic state.

Physiological control during diving is complex. In addition to neural control, a role for hormonal contribution in diving was suggested, based on the observation that adrenal glands continue to be perfused during forced submergence in Weddell seals (Zapol et al., 1979). In freely diving Weddell seals (*Leptonychotes weddellii*), Hochachka and colleagues found that adrenal hormone concentrations (both epinephrine and norepinephrine) increased as a function of dive duration and decreased rapidly during recovery (Hochachka et al., 1995). Besides catecholamines, other hormones, such as thyroid hormones, may be important regulators of the dive response.

Thyroid hormones have widespread physiological and metabolic activity which affects the function of virtually every organ. Thyroid hormones profoundly influence oxidative metabolism in all vertebrate classes and hence their involvement in the setting of metabolic rates in endotherms was proposed (Hulbert, 1986). Hulbert and Else (1981) suggested that the fivefold greater metabolic rate of mammalian tissues compared with reptile tissues was due to a threefold greater thyroxine (T_4_) level in mammals. In humans, basal metabolic rate (BMR) measurements were used historically as a clinical test for evaluating thyroid function (Klein, 2001). The crucial role that thyroid hormones play in regulating metabolism can be seen in humans with abnormal thyroid function; e.g., thyrotoxicosis is associated with an increase in endogenous glucose production, hepatic insulin resistance, and concomitant hyperglycemia (for review see Chidakel et al., 2005). In addition to the profound effects on energy metabolism, thyroid hormones also strongly affect the cardiovascular system. Various mammalian studies demonstrated a strong positive correlation between elevated circulating thyroid hormone levels and resting heart rate (Rutherford et al., 1979; Breisch et al., 1989; Karaus et al., 1989; Hoey et al., 1991; Sernia et al., 1993).

Given the crucial role of thyroid hormones in the regulation of metabolism, hypometabolism during diving may be associated with a reduced thyroid hormone level in divers. In fact, Hochachka speculated that at-sea hypometabolism and metabolic efficiency in seals is likely influenced by thyroid hormone concentrations (Hochachka, 1991). While thyroid hormone concentrations have never been measured during diving, an approach used in other systems is to examine the consequences of hormone excess. A common strategy in thyroid hormone research has been to address specific questions by inducing hyperthyroidism (Hulbert, 2000). The majority of these studies considered the effect of the hormone on the resting state of organisms rather than specific periods of metabolic demand, when energy turnover is likely to be affected by thyroid hormones. Diving is such an example, where animals undergo a variety of metabolic and cardiovascular adjustments. The hyperthyroid state is associated with elevated metabolic rate and elevated heart rate; physiological responses that are opposite to those commonly observed during diving. Hence, if metabolism is increased in hyperthyroid seals, their diving ability would likely be negatively affected. Furthermore, we hypothesized that if hyperthyroidism has functionally the opposite effects on seals as hypothyroidism, then a reduced metabolic rate during diving might be indicative of a hypothyroid state during normal diving.

The present study aimed to investigate the functional importance of hypometabolism during diving in harbor seals. By administering thyroid hormone (T_4_), we intended to offset the hypometabolism in a diving seal. If metabolism in the hyperthyroid state is increased during diving, then oxygen consumption would increase and a decrease in the seal's aerobic dive capacity would likely result. We therefore expected that in order to perform a similar dive pattern, hyperthyroid seals would require a stronger cardiovascular response (i.e., increased bradycardia and peripheral vasoconstriction) when compared to a euthyroid seal and would possibly incur a greater contribution from anaerobic metabolism. Alternatively, seals might end dives prematurely. To investigate these mechanisms, we monitored metabolic rate, heart rate, and post-dive lactate concentrations in euthyroid and hyperthyroid harbor seals performing 5-min dives on command.

## Materials and methods

### Study animals and training

Five captive juvenile harbor seals (*Phoca vitulina richardii*, two males and three females) with a mean body mass of 30 ± 3.5 kg (range: 26–35 kg) were used in this study. Animals were captured at a maximum age of one month (seals 1 and 2 in August 1996 and seals 3–5 in September 1997) and maintained in captivity for about 1 year before being used in the experiments. We designed a carefully directed training regime, based on positive reinforcement, to shape the animal's behavior for the experiments. During diving experiments, seals were never restrained in any way and were free to cooperate or to refuse to perform the dives. By the time the experiments were conducted, the seals had been trained for more than 6 months to perform dives in which they remained stationary underwater for exactly 5 min on command. This procedure, in combination with the young age of the seals and the extended time spent with them, resulted in tame study animals and highly controlled conditions. Experiments started with seals in the euthyroid state and upon completion of these trials, hyperthyroidism was induced and experiments in the hyperthyroid state followed. Experiments for seals 1 and 2 in both euthryroid and hyperthyroid condition were conducted in winter (December 1997 to March 1998), while experiments for seals 3–5 took place during summer (June to September 1998). During this time, air/water temperatures ranged from 5.0/6.1°C in winter to 25.5/17.5°C in summer. All seals gained body mass (M_b_) during the study (mean overall increase: 4.0 ± 1.6 kg) and mass gain rate (kg month^−1^) was not different between thyroid states (*F* = 0.79, *p* = 0.43). All experimental procedures were approved by the UBC Animal Care Committee and were in compliance with the principles promulgated by the Canadian Council on Animal Care.

### Thyroid hormone administration protocol

Hyperthyroidism was induced by administration of the levothyroxine *Synthroid* (Abbott Laboratories, Abbott Park, IL, USA), a synthetic form of Thyroxine (T_4_). The seals were maintained at a dose of 50 μg·kg^−1^·day^−1^, which was administered in tablet form twice daily for up to 6 weeks. To monitor the resulting thyroid hormone concentrations and determine the degree of hyperthyroidism, blood samples were taken twice weekly, 3–5 h after the first levothyroxine dose of the day, using an indwelling extradural vein catheter. Serum concentration of free T_4_ was chosen as the basis for assessment of the thyroid status for the following reasons: (1) T_3_ is primarily an intracellular hormone and its serum concentration fluctuates randomly, making it a less reliable indicator of thyroid status than T_4_ (Feldman and Nelson, 1996); (2) total thyroid hormone levels in plasma (T_3_, T_4_) reflect the levels of thyroid binding plasma proteins rather than the concentration of the biologically active form (free T_3_, free T_4_) (Hulbert and Else, 1999). Hence, free T_4_ was chosen as the physiologically most relevant indicator of thyroid status and was determined using the ACCESS Immunoassay System with the Access free T4 Reagent Pack (Beckman Coulter, Fullerton, CA, USA). The validity of the assay was confirmed by the results of parallelism and recovery of added hormone. The radioimmunoassay results can be affected by species-specific differences in the affinity of binding protein relative to the affinity of the antibody employed and most assays were developed for humans. However, while this could have affected the absolute values determined for the seals, it would have not affected the relative differences observed between treatment groups.

### Respirometry

Metabolic rates of seals were determined measuring oxygen consumption rates (ml O_2_·min^−1^·kg^−1^) using an open flow-through respirometry system. An acrylic dome (volume: ~50 l), floating on the water surface, served as metabolic chamber. Airflow was measured using an electronic mass flowmeter (Omega Engineering, Inc., Stamford, CT, USA). A variable vacuum pump, connected in series with the mass flowmeter, pulled air through the system. A flow of 10 l·min^−1^ kept the fraction of oxygen within the dome above 18.5%. Air was continuously subsampled from the main flow and drawn through two columns filled with a drying agent (Drierite) and a CO_2_ scrubbing agent (Ascarite), prior to determination of oxygen content. The oxygen content was determined by use of a paramagnetic oxygen analyzer (Beckman, Schiller Park, IL, USA). The oxygen analyzer was calibrated before each trial using 99.995% pure N_2_ (PraxAir, Richmond, BC, Canada) and outside air (set to 20.95% O_2_). Ambient temperature and humidity were monitored throughout the trials. Voltage output from the oxygen analyzer and flowmeter were fed into an A/D-converter (DATAQ Instruments, Inc. Akron, OH, USA), and average values were recorded and stored every 5 s onto a desktop computer using WinDaq (DATAQ Instruments Inc.). The system was calibrated using the nitrogen dilution technique and oxygen consumption was calculated with equation 11b in Fedak et al. (1981).

To measure resting metabolic rate (RMR) in water, a seal was directed to place its head into the respirometry dome and oxygen consumption was measured for a minimum of 6 min. A platform 1 m below the dome allowed the seal to remain stationary, without having to spend additional energy for maintaining position when breathing within the dome. During the dive trials a seal submerged on command and remained stationary at the tank bottom, approximately 1.5 m below the surface. Upon completion of a dive, the seal immediately surfaced to breathe into the respirometry dome. Post-dive oxygen uptake was measured while the seal's head remained within the dome for at least 6 min, after which pre-dive levels had been reached. The seal was then instructed to leave the dome, while dome air was continuously sampled until the oxygen concentration reached ambient values.

Oxygen consumption rate was calculated over individual dive cycles (dive and subsequent post-dive surface interval). To this end, the total amount of oxygen removed from the ambient air during the post-dive surface interval was divided by the duration of the dive cycle (Castellini et al., 1992). Dive cycles in our study consisted of a 5 min dive followed by a 6 min surface interval.

### Lactate

To check for possible lactate washout after a dive, blood samples were taken in a separate round of dive trials conducted during the summer months. Three seals (seals 3–5) were trained to station on deck (adjacent to the dive tank) for 15 min immediately after completion of a dive. Using an indwelling catheter positioned in the extradural vein, the first blood sample was obtained within 2 min after a dive. Subsequent samples were taken at 5, 9, 12, and 15 min after surfacing. Blood samples were transferred into vacutainers and kept on ice. The catheter was flushed with saline between each sampling. Without delay the blood was centrifuged and the plasma was removed and kept frozen at −80°C until analysis. Lactate concentration was determined using an assay (modified after Bergmeyer, 1970) for analysis in a photometer (UV/VIS Spectrometer, Model Lambda 2, PerkinElmer, Wellesley, MA, USA). Two replicate measurements were taken from each sample to ensure reproducibility and were compared with standards of known lactate concentration.

### Heart rate

To investigate the effect of thyroid hormone level on heart rate, we recorded the electrocardiogram (ECG) of seals during the dive trials and when resting on land, using a purpose built data-logging system. This system consisted of a modified Tattletale Lite data-logger (Onset Computer Corp., Pocasset, MA, USA) and incorporated a modified Polar OEM ECG amplifier [Polar Electro Inc., Lake Success, NY, USA; for details see: Andrews (1998)]. The entire data-logger assembly was cast in epoxy (Sealtronic Ltd., Burnaby, BC, Canada). Two skin-surface electrodes were attached to each seal. They were positioned anterior (above scapula) and posterior (above hip) to the heart, forming a diagonal line across the dorsal midline. This position resulted in a clean ECG signal even during movement and diving. To fasten the data-logger, two plastic webbing buckles were glued onto the seal (10-min epoxy) which fitted to counterparts attached to the data-logger. This set-up allowed for easy instrumentation of the seals before a trial. We sampled the ECG at a frequency of 100 Hz. At the end of each trial the data-logger could easily be removed and data were downloaded onto a desktop computer.

For analysis data were displayed in AcqKnowledge (BIOPAC Systems Inc., Goleta, CA, USA) to detect the QRS complex, and the mean inter-beat interval (r–r interval) was determined before, during, and after a dive and then converted to heart rate (beats·min^−1^) for surface and submersion periods. Two seconds were omitted at the beginning and end of a dive to avoid rounding errors. To compute a heart rate profile for the dive trials, we averaged heart rate over a 20 s period before a dive (pre-dive), over each 1 min period during a dive, and over 30 s after completion of a dive (post-dive). We furthermore calculated mean dive heart rate for the entire period of submersion, which was also expressed as a percentage of resting heart rate.

### Data collection

Metabolic rate, heart rate, and post-dive plasma lactate concentrations were determined from separate trials. For metabolic rate and heart rate measurements, a minimum of 10 trials per seal were conducted for both the euthyroid and hyperthyroid state. Post-dive lactate concentrations were determined after five dives per seal for both thyroid conditions. Only one successful 5-min dive per seal was conducted each day.

### cADL and maximum observed dive duration

To investigate the effect that a changed thyroid status might have on the aerobic diving capacity of seals, we determined the calculated aerobic dive limit (cADL) for euthyroid and hyperthyroid seals. Total body O_2_ stores for seals were estimated according to Davis et al. (1991, Appendix 2). The cADL was then calculated for each seal and thyroid hormone condition by dividing estimated O_2_ stores by the mean RMR measured for each seal as an approximation of metabolic rate during stationary diving (i.e., without muscular work) (Castellini et al., 1992). In separate trials, four seals were equipped with a purpose-built submergence sensor, mounted to the head, that enabled us to record the duration of spontaneous dives, which they conducted undisturbed in their communal holding tank (16.5 m long × 2 m wide × 1.5 m deep). All four of these seals were monitored during 3–5 trials (each lasting 24–48 h) of spontaneous diving in each thyroid condition. For each trial the single longest dive duration was recorded.

### Statistical analysis

All statistical analyses were conducted using JMP (v.8.0.2.2, SAS Institute Inc.). Differences in free T_4_ levels, oxygen consumption rates ($s\dot{V}o_{2}$), lactate concentrations, heart rate, cADL, and maximum observed dive duration during different thyroid states (euthyroid vs. hyperthyroid) and, where applicable, during different activities (resting vs. diving) were tested using a linear mixed-effects model (standard least squares regression fitted by REML). Thyroid status and activity were included as fixed effects, while seal ID was included as a random effect. To test for significance of the correlation between diving heart rate and post-dive blood lactate concentration, the Pearson Product Moment Correlation test was used. Significance was accepted at the level of *p* < 0.05. Data are presented as means ± 1 standard deviation (mean ± SD).

## Results

### Experimentally induced hyperthyroidism

**Figure 1:**
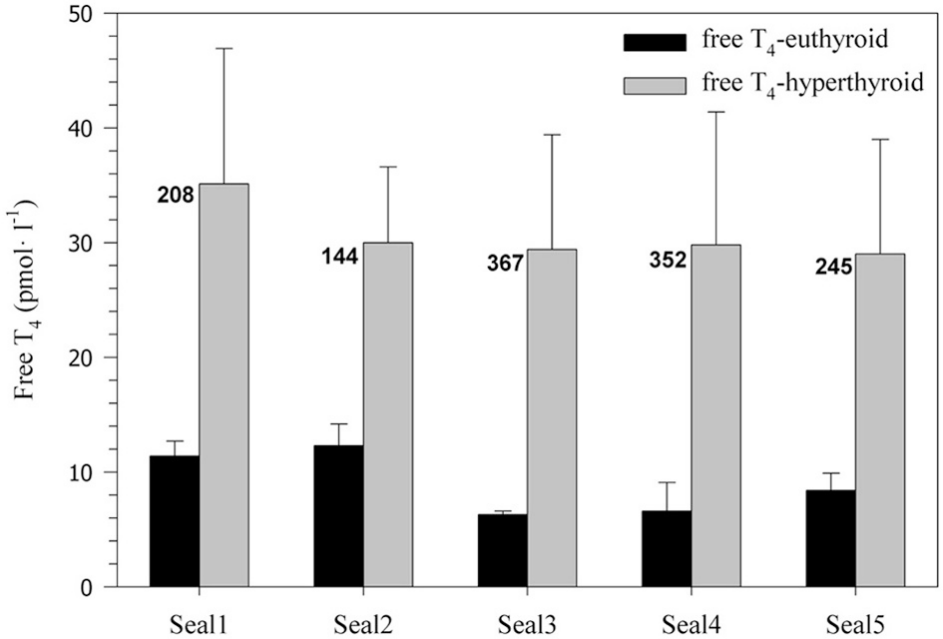
**Free serum T_4_ concentration (pmol·l^−1^) before and after experimentally induced hyperthyroidism in five harbor seals (values are means ± SD; *n* = 6 samples per seal and condition).** Numbers beside the bars indicate the percent increase in free T_4_ levels associated with levothyroxine treatment.

Administration of levothyroxine (50 μg·kg^−1^·day^−1^) resulted in a sustained and significant 2.5–4.5-fold increase in serum free T_4_ levels in all harbor seals (31 ± 11.1 pmol·l^−1^) when compared with the euthyroid control state (9 ± 2.7 pmol·l^−1^; *F* = 77.40, *p* < 0.0001; Figure 1). Euthyroid free T_4_ levels were greater in seals 1 and 2, which were measured during the winter months and the relative increase in free T_4_ after administration of levothyroxine was lower than in seals 3–5 measured during summer (Figure 1).

### Metabolic rate

**Figure 2:**
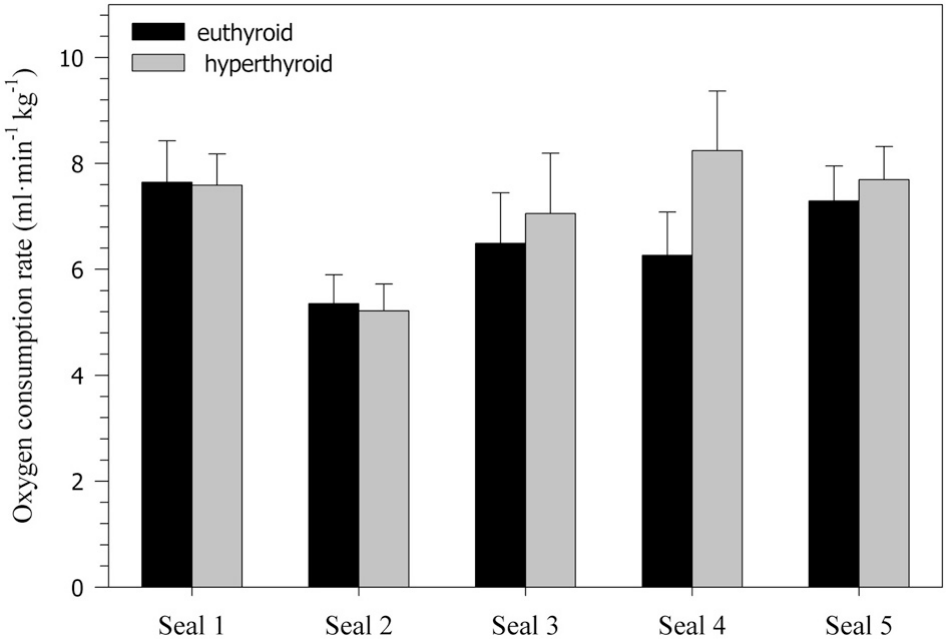
**Oxygen consumption rates (ml·min^−1^·kg^−1^) during diving in five euthyroid and hyperthyroid seals (values are means ± SD; *n* = 10 trials per seal and condition)**.

Oxygen consumption rate ($s\dot{V}o_{2}$) was significantly affected by both thyroid status (*F* = 18.79, *p* < 0.0001) and activity (*F* = 229.78, *p* < 0.0001). During diving, $s\dot{V}o_{2}$ was significantly reduced by about 25% in both the euthyroid (*F* = 162.60, *p* < 0.0001) and hyperthyroid state (*F* = 86.38, *p* < 0.0001) when compared with resting at the surface of the water (Table 1). In the hyperthyroid state, seals had a significantly greater $s\dot{V}o_{2}$ (~7–8%) during both resting (*F* = 7.11, *p* < 0.01) and diving (*F* = 13.21, *p* < 0.001) than in the euthyroid state (Table 1, Figure 2). As with most variables investigated, the response by seals varied between individuals. For example $s\dot{V}o_{2}$ during diving in seals 1 and 2, measured during winter, did not differ with thyroid state, while it did vary in seals 3–5 (Figure 2).

**Table 1:** **Oxygen consumption rates ($s\dot{V}o_{2}$) in the euthyroid and hyperthyroid state**.

**Activity**	**Oxygen consumption rates**		
	**Euthyroid (ml·min^−1^·kg^−1^)**	**Hyperthyroid (ml·min^−1^·kg^−1^)**	**% increase in hyperthyroid state**
Resting	8.83 ± 1.05	9.42 ± 1.16^†^	6.7^†^
Diving	6.61 ± 0.90^*^	7.16 ± 1.16^*^^†^	8.3^†^

Values are grand means which were established from individual seal means (N = 5 seals, n = 10 trials per seal and condition).

* Indicates a significant difference from the resting situation and

† indicates a significant difference from the euthyroid state.

### Lactate

**Figure 3:**
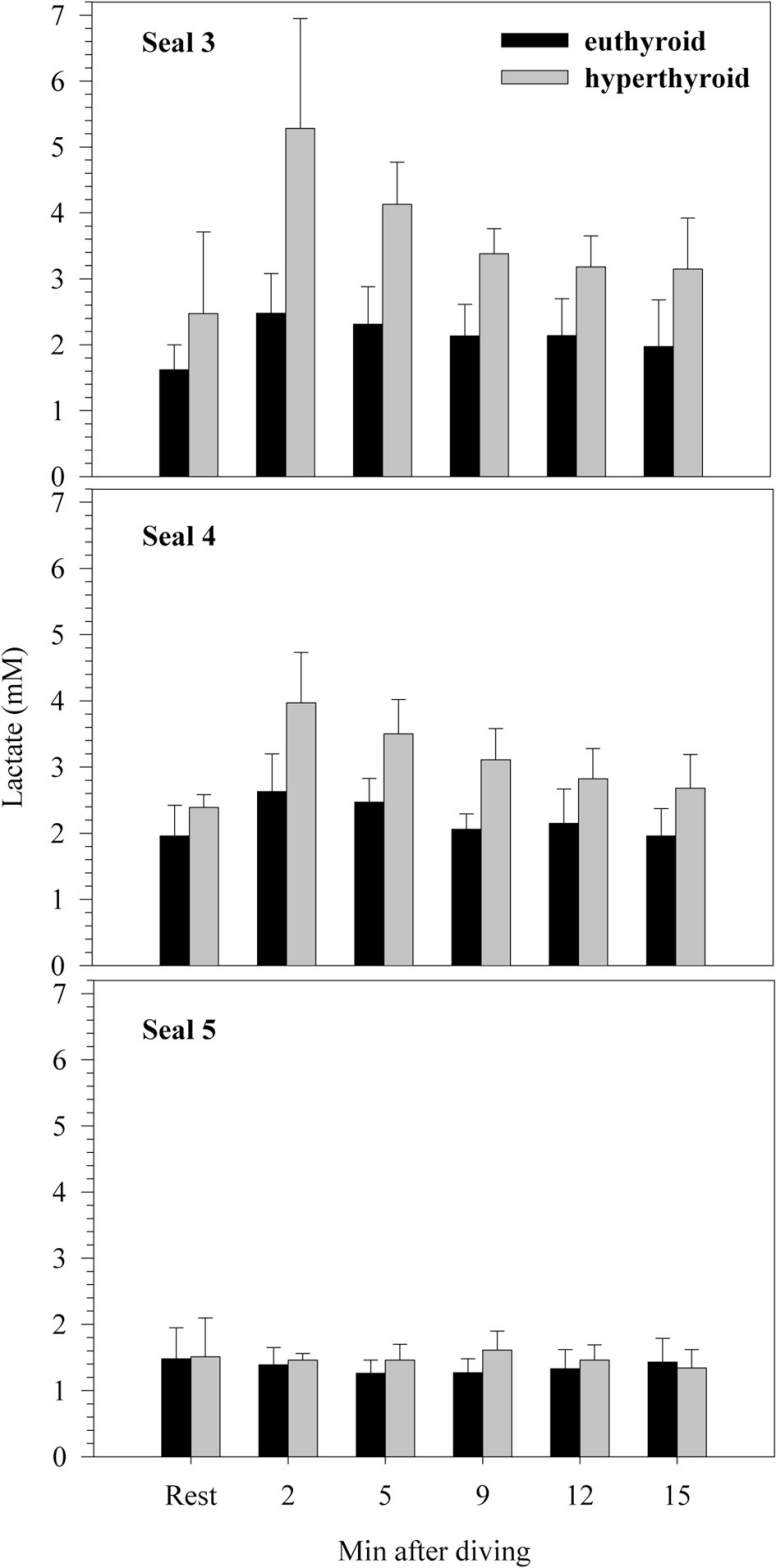
**Resting and post-dive lactate concentrations (mM) for three individual harbor seals (values are means ± SD; *n* = 5 trials per seal and condition)**.

There was no significant difference in resting blood lactate concentration between the euthyroid and hyperthyroid condition (1.68 ± 0.25 vs. 2.12 ± 0.53 mM; *F* = 4.17, *p* = 0.05; Figure 3), but in two of the three harbor seals, resting lactate levels were elevated in the hyperthyroid condition. After a 5 min dive in the euthyroid state, blood lactate concentration was no different from resting [La]. However, in two out of three seals, post-dive lactate concentrations were distinctly elevated in the hyperthyroid state. This response was consistent throughout the five trials conducted with each seal (Figure 3). Accordingly, mean post-dive lactate concentrations in the hyperthyroid condition were significantly greater than in the euthyroid condition over the time-course observed (*F* = 121.40, *p* < 0.0001).

### Heart rate

**Figure 4:**
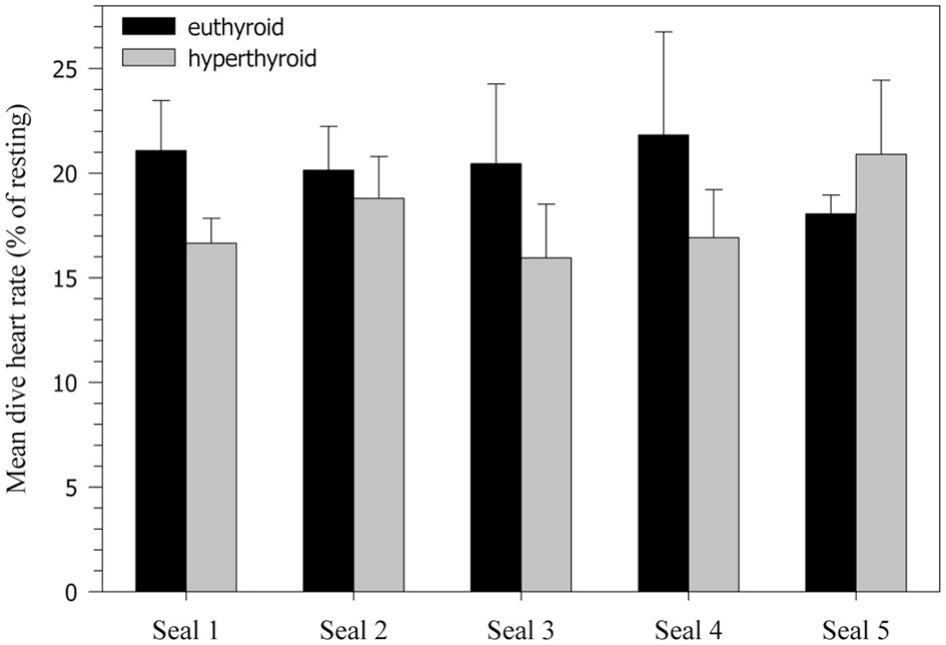
**Heart rate decline during 5 min dives (% of resting heart rate).** Values are means ± SD (*N* = 5 seals, *n* = 10 trials per seal and condition).

Heart rates of resting harbor seals in the euthyroid state (hauled out on deck) averaged 114.3 ± 4.0 beats·min^−1^. In the hyperthyroid state resting heart rates were significantly elevated by about 5% (119.6 ± 4.2 beats·min^−1^; *F* = 7.15, *p* < 0.01), when compared with the euthyroid state. During the dive trials the following general heart rate pattern was observed in both euthyroid and hyperthyroid states (Table 2): Heart rate was elevated before submergence (~10–20% above resting) and dropped immediately upon submergence to less than 40 beats·min^−1^ (~30% of resting value). Heart rate then continued to fall throughout the dive to a minimum of 16 beats·min^−1^. Upon emergence heart rate increased to a level of ~20–25% above resting. Mean dive heart rate was significantly lower in the hyperthyroid state and, hence, diving bradycardia was more pronounced during experimental hyperthyroidism (*F* = 13.98, *p* < 0.001; Figure 4), when compared with the euthyroid state. Throughout the various phases of submergence (min 1–5 of submergence), diving heart rates in the hyperthyroid state were significantly lower than in the euthyroid state, while heart rates before and after submergence did not differ significantly (Table 2). Furthermore, mean dive heart rate was significantly negatively correlated with post-dive lactate concentration in three seals in the hyperthyroid state (Pearson's correlation *coefficient* = −1, *p* = 0.003). A lower heart rate during diving in these seals was associated with a greater post-dive lactate concentration.

**Table 2:** **Heart rates before, during, and after submergence in the euthyroid and hyperthyroid state**.

**Phase of dive cycle**	**Euthyroid (beats·min^−1^)**	**Hyperthyroid (beats·min^−1^)**	**Hyperthyroid (% of euthyroid)**
Pre-dive	133.35 ± 5.09	130.76 ± 7.38	98.1
Dive: min 1	31.76 ± 4.18	29.23 ± 6.01^†^	92.0^†^
Dive: min 2	27.61 ± 3.38	25.39 ± 4.71^†^	91.9^†^
Dive: min 3	22.39 ± 2.13	20.43 ± 5.29^†^	91.2^†^
Dive: min 4	18.23 ± 3.46	17.41 ± 4.21	95.5
Dive: min 5	16.60 ± 3.86	15.62 ± 3.06^†^	94.1^†^
Post-dive	144.72 ± 8.00	145.76 ± 5.12	100.7

Values are grand means which were established from individual seal means (N = 5 seals, n = 10 trials per seal and condition.

† Indicates a significant difference from the euthyroid state.

### cADL and maximum observed dive duration

As each animal continued to grow during the experimental period, the M_b_ of seals was greater during the hyperthyroid trials when compared with the euthyroid trials (Table 3). Accordingly, estimated body O_2_ stores for seals were significantly greater during hyperthyroid trials (Table 3; *F* = 8.73, *p* = 0.04). However, because RMR increased in hyperthyroid seals (Table 1), their cADL was significantly reduced when compared with euthyroid seals (Table 3; *F* = 10.91, *p* = 0.03). Maximum dive duration observed during spontaneous diving activity was considerably and significantly shorter in hyperthyroid seals (Table 3; *F* = 55.68, *p* < 0.01).

**Table 3:** **Estimated total body O_2_ stores, cADL, and maximum observed dive duration for seals in the euthyroid and hyperthyroid state**.

**Seal**	**M_b_(kg)**	**O_2_ store (ml)**	**cADL (min)**	**Maximum dive duration (min)**
**EUTHYROID**				
Seal 1	32.2	1831.1	6.52	4.68
Seal 2	35.9	2037.5	7.86	7.49
Seal 3	30.0	1702.2	6.20	5.03
Seal 4	26.3	1495.9	6.35	7.35
Seal 5	30.9	1753.8	5.61	
Grand mean	31.1 ± 3.5	1764.1 ± 196.9	6.51 ± 0.83	6.14 ± 1.49
**HYPERTHYROID**				
Seal 1	33.6	1908.5	5.69	2.42
Seal 2	36.8	2089.0	7.54	4.72
Seal 3	30.9	1753.8	6.07	2.32
Seal 4	28.6	1624.8	5.92	3.25
Seal 5	30.9	1753.8	5.35	
Grand mean	32.1 ± 3.1^†^	1826.0 ± 178.1^†^	6.11 ± 0.85^†^	3.18 ± 1.11^†^

*O*_2_ stores were calculated according to Davis et al. (1991).

cADL was calculated by dividing estimated O_2_ stores by the mean RMR measured for each seal as an approximation of metabolic rate during stationary diving. Values for max. dive duration are means established from 3–5 trials (lasting 24–48 h), during which the single longest spontaneous dive was recorded.

† Indicates a significant difference from the euthyroid state.

## Discussion

### Effects of elevated thyroid hormone concentration on RMR

We found a significant increase in RMR of harbor seals after administration of T_4_. However, the increase in metabolic rate observed in our study was surprisingly low when compared with the large response to experimental hyperthyroidism typically observed in terrestrial animals (Hoch, 1974; Dauncey, 1990). An insufficient degree of hyperthyroidism can be ruled out as an explanation for the relatively small changes in metabolic rate and heart rate in the hyperthyroid state as levothyroxine administration significantly elevated free T_4_ levels (to 2.5–4.5 times the euthyroid level; Figure 1) for a prolonged period. The effects of experimentally elevated thyroid hormone levels on metabolic rate have never been investigated in pinnipeds, but a number of pinniped studies suggest a strong correlation between RMR and thyroid hormone levels. RMR in immature, growing pinnipeds is elevated when compared with adult animals and this is generally attributed to greater energetic requirements associated with the formation of new tissue and higher thermoregulatory costs (Hart and Irving, 1959; Miller and Irving, 1975; Rea and Costa, 1992). In those studies on harbor seals and northern elephant seals, RMR slowly decreased as the animals approached maturity. Other studies reported increased thyroid activity in young southern elephant seals (Little, 1991; Bryden, 1994), harp seals, (*Phoca groenlandica;* Leatherland and Roland, 1979), gray seals (*Halichoerus grypus*; Engelhardt and Ferguson, 1980), and harbor seals compared with adults (Stokkan et al., 1995; Woldstad and Jensen, 1999), which coincides with the elevated RMR observed in juvenile pinnipeds. Natural variations in the amount of plasma thyroxine were reported to be correlated with changes in the RMR of adult harbor seals (Ashwell-Erickson and Elsner, 1981) and gray seals (Boily, 1996). Ashwell-Erickson et al. (1986) investigated the relationship between metabolism and thyroid hormone concentration during molt in harbor and spotted (*P. largha*) seals. They observed a concurrent decrease in RMR and thyroid hormone concentration at the onset of molt, which would serve to decrease food requirements at a time when seals have to stay out of the cold water. Once hair regeneration was well underway, thyroid hormone concentrations increased to their maximum, and RMR rose to its pre-molt level. In juvenile seals, metabolic rates during the annual molt fluctuated by ±15%, concurrent with free T_4_ fluctuations (Ashwell-Erickson et al., 1986). Although the observed increase in metabolic rate during hyperthyroidism was modest, these results support the claims that fluctuations in RMR in seals are at least in part driven by changes in thyroid hormone levels.

There are several possible explanations for the limited magnitude of the increase in metabolic rate during hyperthyroidism in our harbor seals. (1) Seals might be able to down-regulate their sensitivity to thyroid hormones. Hochachka et al. (1991) suggested that high-altitude adapted humans did have a decreased sensitivity to thyroid hormones, which could increase their muscle efficiency in the face of hypoxia. Similarly, a down-regulated sensitivity to thyroid hormones could explain the small increase in harbor seal RMR we observed, even at substantially elevated plasma concentrations of free T_4_. (2) The limited effect of thyroid hormones on harbor seal RMR could be due to potential differences in cell membranes between terrestrial and diving mammals. In general, thyroid hormones increase the permeability of cell membranes through various mechanisms. The resulting leakier membranes require more energy for ion pumping to maintain the ionic integrity of cells (Hulbert and Else, 1981). In diving animals, however, the permeability of cell membranes also determines hypoxia sensitivity. Hochachka and co-workers compared hypoxia resistance and diving ability and found that mitochondria from seal livers were more successful in surviving hypoxia when compared with liver mitochondria of terrestrial animals (Hochachka et al., 1988). In addition, cell membranes of hypoxia-tolerant liver tissue in seals were less permeable than those in the hypoxia-sensitive brain (Hochachka et al., 1988). This reduction in cell membrane permeability would reduce metabolic rate and might therefore be an important mechanism for extending hypoxia tolerance in diving animals (Hochachka and Guppy, 1987). A reduction in cell membrane permeability, by a yet unknown mechanism, could reduce the sensitivity of cell membranes to thyroid hormones in seals and could explain the relatively small increase in RMR observed in our harbor seals after T_4_ administration.

### Hypometabolism during diving

Oxygen consumption and heart rate in both the euthyroid and hyperthyroid state were significantly reduced during diving when compared with resting (Tables 1 and 2), indicating that metabolic suppression occurred during diving. Metabolic rate was decreased by about 25% during diving, when seals lay motionless on the bottom of the tank. This is similar to what was reported for northern elephant seals diving under comparable conditions (Webb et al., 1998). The presence of hypometabolism during natural diving has been suggested in other pinniped studies (Guppy et al., 1986; Le Boeuf et al., 1988; Hindell et al., 1992). The degree of hypometabolism typically observed in these seals varies according to their dive behavior (i.e., resting underwater *vs*. exercise). Castellini et al. (1992) measured oxygen consumption in freely diving Weddell seals conducting dives of up to 82 min and found that metabolic rate during active diving under the ice declined with increasing dive duration, but was not significantly different from resting values. Our seals were not exercising, but rather simply laying on the bottom of the tank, which may have allowed them to reduce metabolic rate below the resting level.

### Hyperthyroid diving metabolic rate

Oxygen consumption while diving when the seals were hyperthyroid was significantly greater (~7–8%) than when they were euthyroid (Table 1, Figure 2). Hence, the ability to reduce metabolism during diving was impaired in the hyperthyroid state. Hyperthyroid post-dive plasma lactate concentrations were significantly elevated in two of the three seals when compared with euthyroid post-dive levels (Figure 3). This illustrates that at least in these two seals there was a net contribution from anaerobic pathways to overall diving metabolism. If lactate accumulated during a dive is not completely metabolized during the following surface period, post-dive oxygen consumption will not accurately reflect the metabolic energy expended during that dive. Kooyman et al. (1980) showed that Weddell seals need up to 1 h to process the lactate produced during extended dives. Consequently, hyperthyroid seals in our study might not have processed all the lactate produced during a dive within the 6 min spent post-dive inside the dome. This is supported by lactate levels that were still elevated 15 min after completion of a dive in two of the three hyperthyroid seals (Figure 3). Hence, diving metabolic rate in the hyperthyroid state might have been even higher than measured in our study.

In the present study we attempted to experimentally shorten the ADL of seals by increasing diving metabolic rate via an induced hyperthyroid state. Maximum dive duration during spontaneous diving activity in hyperthyroid seals was about half of that observed in euthyroid seals (Table 3), suggesting that aerobic dive capacity was significantly altered during hyperthyroidism. Accordingly, we estimated the cADL of our harbor seals to be 6.5 min in the euthyroid state and 6.1 min in the hyperthyroid state (Table 3). In agreement with the estimated cADL of 6.5 min for euthyroid seals, we found no evidence of a net anaerobic contribution to their diving metabolism. By contrast, post-dive lactate concentrations were significantly elevated in two hyperthyroid seals (Figure 3), in which the experimental dive duration (5 min) was closer to their cADL (mean cADL for seals 3–5 was 5.8 min). Hence, the increased oxygen consumption during diving as a consequence of their hyperthyroidism might have pushed seals toward their aerobic limit and triggered an increase in anaerobic metabolism. However, the lactate response was not consistent in all individuals. This could suggest that the experimental dive duration of 5 min is within a transition zone where seals increasingly rely on anaerobic metabolism, or that diving metabolic rate, even when resting on the bottom, is not fixed but can vary both between and within individuals.

Numerous studies demonstrated a correlation between the amount of lactate accumulated in the blood and the level of exercise during a dive. For example, in bottlenose dolphins (*Tursiops truncatus*), lactate concentration after a 5 min stationary breath-hold at the surface was elevated but consistently lower than after active dives of comparable duration (Williams et al., 1999). Similarly, lactate concentration in beluga whales (*Delphinapterus leucas*) was elevated to about three times the resting value after both active swim trials and deep dives, while there was little increase after sedentary breath-holds near the surface (Shaffer et al., 1997). This suggests that locomotor effort during active dives in the above studies increased metabolic costs beyond what could be supplied by aerobic means and necessitated a net contribution from anaerobic pathways.

The increased lactate concentrations we observed in two hyperthyroid seals after 5 min dives is remarkable because it did not result from increased muscular activity. During all dives seals remained stationary and motionless at the bottom of the tank. Hence, the increase in metabolic rate caused by hyperthyroidism was sufficient to require energy production beyond the aerobic capacity during these dives. Alternatively, the increased post-dive lactate levels during hyperthyroidism could have been due to the effect of increased thyroid hormones on glucose turnover. Hyperthyroidism is known to be associated with increased gluconeogenesis and insulin resistance (Chidakel et al., 2005). However, steady-state lactate concentrations are not usually elevated during hyperthyroidism, at least in fed animals (c.f. Sugden et al., 1990), and although two of our three seals did have increased resting lactate, the difference was not statistically significant, whereas post-dive lactate was significantly increased. Furthermore, our supposition that the increase in post-dive lactate was due to an increased rate of oxygen store depletion during hyperthyroid diving is supported by the lower diving heart rate and negative correlation between diving heart rate and post-dive lactate, as explained below.

### Hyperthyroid heart rate

Perhaps the most interesting finding in the present study was the stronger bradycardic response during diving in the hyperthyroid condition, when compared with the euthyroid condition (Table 2, Figure 4). Based on studies in terrestrial mammals, in which thyroid hormone stimulation caused elevated heart rates (tachycardia) (Palacios et al., 1979; Rutherford et al., 1979; Williams et al., 1980; Karaus et al., 1989), one might have expected that heart rates would also be elevated in diving hyperthyroid harbor seals. However, as we predicted, seals frequently displayed significantly lower diving heart rates in the hyperthyroid condition when compared to euthyroid diving heart rates. The seals did appear to have two different, albeit persistent, strategies to cope with hyperthyroidism. One individual did display an increased heart rate during diving in the hyperthyroid state. In contrast, the other four seals exhibited a more profound bradycardia during diving in the hyperthyroid state (Figure 4). This suggests that in the hyperthyroid condition, at least some of our seals might have been pushed toward their aerobic limit, so that a more pronounced diving response became necessary to conserve oxygen. Similar cardiovascular responses to increased metabolic requirements during diving, as a consequence of stress or prolonged dive duration, have been shown in seals (Fedak, 1986) and are of crucial importance for the conservation of oxygen for the hypoxia-intolerant tissues of the heart and brain.

In our study, in both the euthyroid and hyperthyroid condition, seals developed a pronounced bradycardia during 5 min dives and the time course of this heart rate decline was comparable, despite differences in thyroid hormone concentrations (Table 2, Figure 4). The absence of elevated diving heart rates in our hyperthyroid seals, despite an increased level of thyroid hormones, clearly suggests that the dive response was able to override any direct hormonal effect on heart rate.

We found a strong correlation between the degree of diving bradycardia and post-dive lactate production during 5 min dives during hyperthyroidism. The highest post-dive blood lactate values occurred after dives during which heart rate was lowest. Assuming that blood pressure is maintained, a heart rate decline during diving is indicative of increased vasoconstriction. The stronger the vasoconstriction, the more likely contributions from anaerobic metabolism become in under-perfused tissues, resulting in lactate production and, hence, increased post-dive lactate concentrations.

### Individual variability and seasonal effects

Responses in all parameters often varied between individuals, suggesting that there is no standard response of a harbor seal to any given experimental condition. Each seal responded somewhat differently to the challenge of diving and elevated thyroid hormones. Some of the variation in the observed responses might be related to differences in environmental conditions (i.e., summer vs. winter) and the corresponding seasonal changes in natural hormone levels and ambient temperatures. A number of studies demonstrated seasonal variations in thyroid hormone concentrations of seals, often in association with molt (Ashwell-Erickson et al., 1986; Renouf and Brotea, 1991; Boily, 1996). The scope of seasonal variation in free T_4_ levels is typically smaller in adult seals than in juveniles. For example, free T_4_ during molt increased on average between 14 and 46% in sub-adult and adult harbor seals (Renouf and Brotea, 1991) and by ~12% in adult gray seals (Boily, 1996). By contrast, in juvenile harbor and gray seals free T_4_ levels doubled during molt (Renouf and Brotea, 1991; Boily, 1996). In our study, free T_4_ levels in euthyroid seals 1–2 (measured during the winter) were ~67% greater than in euthyroid seals 3–5 (measured during the summer), indicating similar seasonal variation in these juvenile seals (Figure 1). However, after levothyroxine administration free T_4_ levels in all seals were greatly increased (on average by ~263%; Figure 1) and well beyond the scope of any natural seasonal fluctuation. Hence, the contribution of seasonal changes in free T_4_ levels to the observed variation in physiological responses was most likely limited. There may have been a slight seasonal difference in sensitivity to hyperthyroidism as only the three summer seals (Seals 3–5) showed an increase in diving oxygen consumption in the hyperthyroid state (Figure 2), but the mean percent increase for these 3 seals (15.2%) was still surprisingly small given their very large (321%) increase in free T_4_ levels.

Differences in individual behavior and cooperation during experimentation but also in diving capacity (defined as the maximum observed dive duration) may have further contributed to the observed variability in physiological responses, which was most evident during the diving experiments. This illustrates the importance of taking into account the physiological diversity that exists within animal populations when investigating the physiological mechanisms associated with diving. The variability observed in our seals as they dealt with the increased diving metabolism due to induced hyperthyroidism emphasizes the plasticity of physiological responses. It furthermore underlines the ability of air breathing, diving vertebrates to successfully adapt to a variety of challenging situations.

## Conclusions

Perhaps most surprising was the observation that hyperthyroidism did not induce a more pronounced change in the physiology of resting harbor seals. Although both metabolic rate and heart rate were increased in the hyperthyroid state, this increase was small in comparison to the increase reported during experimental hyperthyroidism in terrestrial vertebrates. A possible explanation for the weak response to elevated T_4_ might be a decreased sensitivity of diving animals to the action of thyroid hormones. Another striking result is the occurrence of elevated lactate concentrations after a 5 min dive in some seals, when diving in the hyperthyroid state. We measured a significant elevation in oxygen consumption during diving in the hyperthyroid condition (~7–8%), when compared with the euthyroid control condition. Overall metabolic rate (aerobic plus anaerobic) during diving might have been even higher in the hyperthyroid condition, as indicated by the elevated post-dive lactate concentration. Most interestingly, increased metabolic rate during diving in the hyperthyroid state was accompanied by a decreased heart rate, suggesting that the higher metabolic rate required a stronger cardiovascular response to enable seals to conserve oxygen and stay submerged for the requested duration. To unequivocally illustrate this chain of events, a larger sample size than was possible in our study would be advantageous. Similarly, for a clear determination of the critical dive time, beyond which metabolic adjustments during hyperthyroidism are required, it would be desirable to conduct trials with multiple dive durations, covering a greater range than our study. Nevertheless, our results demonstrate the powerful role of thyroid hormones in metabolic regulation and support the notion that they might be instrumental in modulating the at-sea metabolism of phocid seals.

### Conflict of interest statement

The authors declare that the research was conducted in the absence of any commercial or financial relationships that could be construed as a potential conflict of interest.
